# A Micro‐CT Based Cadaveric Study Investigating Bone Density Changes During Hip Arthroplasty Surgery

**DOI:** 10.1002/jor.26032

**Published:** 2024-12-26

**Authors:** Vineet Seemala, Mark A. Williams, Richard King, Sofia Goia, Paul F. Wilson, Arnab Palit

**Affiliations:** ^1^ WMG University of Warwick Coventry UK; ^2^ Department of Trauma & Orthopaedics University Hospitals Coventry and Warwickshire NHS Trust Coventry UK

**Keywords:** bone density, density calibration phantom, micro computed tomography (μCT), total hip replacement, uncemented implants

## Abstract

The impact of broaching and uncemented implantation on bone density during total hip arthroplasty (THA) remains unclear. Previous studies have typically examined extracted bone sections, which may not directly correlate with outcomes in human hip systems. This study aimed to evaluate bone density changes resulting from broaching and uncemented implantation using micro‐computed tomography (μCT) on cadaveric samples. An in‐house density calibration phantom (DCP) was developed by validating the densities of polymer inserts through mass and volume measurements. Its performance was then evaluated using lamb bone in comparison with a commercial DCP (QRM‐50124). The sensitivity of density predictions to μCT scan parameters was also evaluated with the lamb bone. Additionally, density predictions from medical‐CT and μCT scans were compared using the in‐house DCP. Finally, uncemented THA procedures were performed on three cadaveric femurs, each undergoing three μCT scans at various surgical stages to assess changes in bone density. The density predictions obtained using the in‐house DCP achieved an accuracy of ±0.097 g/cc compared to QRM‐50124, with a precision of ±0.052 g/cc. The sensitivity to changes in μCT scan parameters was ±0.022 g/cc. Notably, density predictions from medical‐CT and μCT scans were similar, particularly in cortical bone. Broaching and implantation led to an average increase in bone density of 0.137 g/cc, which was attributed to the accumulation of bone debris around the bone‐implant interface. This accumulation raised the bone volume fraction, ranging from 3.31% to 20.69%, which acts as an autograft. These measurements have been made for the first time using a µCT and an in‐house DCP.

## 1 Introduction

Total Hip Arthroplasty (THA) is an effective surgery for relieving pain and restoring mobility in patients with hip osteoarthritis. Hip implants are categorised into cemented and uncemented, based on their bonding mechanisms. Uncemented implants rely on mechanical press fitting for primary stability and osseointegration for secondary stability [1, 2, 3, 4, 5]. Although modern cemented implants perform well, limitations like poor tensile strength and the risk of osteolysis have led to increased use of uncemented implants [6, 7]. In the UK, the use of cemented hips nearly halved from 2006 to 2021, while uncemented implants increased by almost 2.5 times during the same period [8]. By 2030, the number of THA in young adults is expected to increase fivefold [9], with over 80% of these patients receiving uncemented implants [8]. Therefore, it is important to evaluate uncemented prostheses to minimise the risk of surgical complications.

To ensure proper press‐fit and reduce the risk of periprosthetic fractures with uncemented implants, bone density is crucial [10, 11, 12]. Preparation of the cavity before implantation involves broaching, resulting in osseodensification by breaking and compacting trabeculae within the bone tissue. Different types of broaches, such as compaction broaches, blunt extraction broaches, and sharp extraction broaches, are used in this process. Despite variations in broach design, all contribute to osseodensification [13]. This process enhances primary implant stability by reducing micromotion and improving fixation strength before osseointegration [7, 14, 15]. However, bone densification around implants and the effect of surgical intervention (broaching and implantation) on bone density have not been thoroughly evaluated. Some studies, using mechanical setups to mimic broach/implant surfaces, found that different surface finishes and higher initial bone density increased bone densification [13, 14, 16]. However, these studies did not replicate the actual bone‐implant interface. Furthermore, these studies were performed using cadaveric femur samples and medical‐CT scans [14] or bovine bone samples using μCT scans [13]. This limits their applicability due to the resolution limitations of medical‐CT, which cannot differentiate trabecular bone structure, making it difficult to quantify the breaking and compaction of trabecular bone. Furthermore, the use of μCT scans in bovine bone samples reduces their relevance to human hip applications. The evaluation of changes in bone density due to surgical intervention using μCT scans of cadaveric human samples to obtain more in‐depth information is lacking in the literature.

Current literature typically employs commercial density calibration phantoms (DCPs) in medical‐CT scans to correlate CT scan intensity with density values. These commercial DCPs contain hydroxyapatite in their inserts to mimic bone material, making them costly. Additionally, these phantoms are designed for specific use in either medical‐CT or μCT scanners. To the best of the authors' knowledge, there is no existing development or validation of an in‐house DCP that can be used cost‐effectively for both medical‐CT and μCT scans. Furthermore, the comparison of density predictions between the two CT scan modalities, is absent in the literature.

Bone density estimation is crucial for developing personalised finite element analysis (FEA) of biomechanical systems, rather than relying on a generalised FEA that uses population‐averaged bone material properties. This is because material constants correlate with bone density through empirical relationships [17, 18], and most of these correlations follow a power law. Bone density estimation is particularly important when analysing changes due to broaching and implantation steps, as these affect bone integrity and fracture risk.

Therefore, the aim of the study was to investigate the change in bone density resulting from the broaching operation and uncemented implantation through μCT based cadaveric study. To address the study's aim, the following objectives were established: (a) To develop an in‐house DCP for mapping CT scan intensities to bone density, validate the results, and investigate its prediction sensitivity. (b) To compare density predictions between medical‐CT and μCT scans using the developed mapping procedure. (c) To examine changes in bone density due to the broaching operation and uncemented implantation by performing THA on three cadaveric femurs and conducting μCT scans at various surgical stages. It is worth mentioning that the density measured in this study refers to real density of bone.

The manuscript is organised as follows: the development of the in‐house DCP is explained, followed by the validation of density predictions using the in‐house DCP compared to a commercial DCP with a lamb bone. The sensitivity of the density prediction to the µCT scan parameters is evaluated using the lamb bone. Additionally, the difference in density of the cadaveric femur between the two CT scan modalities, medical‐CT and µCT, is assessed. The experimental study on performing uncemented THA is described, along with the evaluation of changes in bone density due to uncemented THA at intermediate surgical steps using µCT.

## 2 Methods

### 2.1 Development and Validation of In‐House Density Calibration Phantom (DCP)

An in‐house DCP was developed using five polymer inserts (nylon, PEEK, Acetal, PPS, PTFE) with densities of 1.14 g/cc, 1.32 g/cc, 1.42 g/cc, 1.64 g/cc, and 2.2 g/cc, respectively, according to the manufacturer's specifications. These materials were selected to closely match the bone density reported in previous studies [19, 20], covering both trabecular and cortical bone, while minimising CT artifacts. The densities of the inserts were validated by determining their volumes through two methods: μCT scanning and laser scanning. The μCT scanning was performed using a Zeiss Metrotom 1500 at 60 kV, 650 μA, with a 1000 ms integration time and an isotropic voxel size of 48.25 µm. In addition, laser scanning was conducted with a Nikon ModelMaker H120, mounted on a Nikon MCAx S portable CMM arm, achieving a minimum resolution of 35 µm and a combined accuracy of 32 µm (2σ) with the scanning arm. The masses were measured with a Mettler Toledo analytical balance (readability: 0.0001 g), and subsequently the densities were calculated to verify the manufacturer‐specified values.

#### 2.1.1 Density Mapping Procedure

The segmentation of the DCP inserts from each CT scan was performed by establishing a threshold for each insert in Avizo 3D 2021 (Thermo Fisher Scientific, Germany). To determine the mean intensity of each DCP insert, an intensity histogram was generated, and the mean intensity was computed. To establish the relationship between CT intensity and density, a linear regression line was determined between the mean intensities and densities of the DCP inserts using Matlab 2022b (The MathWorks Inc., Natick, MA). The density was finally assigned based on the intensity values at each voxel, utilising the density calibration line determined for a specific scan.

#### 2.1.2 Validation of the Density Prediction

The accuracy of the density prediction using the in‐house DCP was validated by μCT scanning of a lamb bone in conjunction with the in‐house DCP and a commercially available DCP (QRM‐50124, QRM GmbH, Moehrendorf, Germany). The scan parameters were as follows: Tescan Unitom XL 170 kV, 70 W, 250 ms integration time, 100 µm isotropic voxel size, 0.25 mm Cu filter. The accuracy of the density prediction using the in‐house DCP was evaluated by comparing the lamb bone density predictions from the in‐house DCP and the commercial DCP, finding a correlation between the two. Furthermore, the precision of the density prediction using the in‐house DCP was evaluated by calculating the standard deviation of the difference in the density measurements of the lamb bone from two subsequent μCT scans performed with the same scan parameters without any interventions.

#### 2.1.3 Sensitivity of the Density Prediction

The sensitivity of the density prediction using the in‐house DCP with different µCT scan parameters was evaluated. A lamb lower limb, stripped of soft tissues and stored at −20°C, was thawed to room temperature for experimentation. A full factorial design (DOE) was employed to investigate CT parameter effects on predicted density, with voltage and exposure tested at two levels each and filtration at three levels. The lamb femur, in conjunction with the in‐house DCP, underwent μCT scanning on a Tescan Unitom XL with a fixed 70 μm isotropic voxel size, while systematically varying voltage, exposure, and filtration settings as detailed in Table 1.

**Table 1:** Sensitivity study design of experiments (DOE).

Scan	Voltage (kV)	Exposure (ms)	Filter
Scan 1	100	80	no
Scan 2	100	80	0.25 mm Cu
Scan 3	100	80	0.25 mm Sn
Scan 4	100	100	no
Scan 5	100	100	0.25 mm Cu
Scan 6	100	100	0.25 mm Sn
Scan 7	110	80	no
Scan 8	110	80	0.25 mm Cu
Scan 9	110	80	0.25 mm Sn
Scan 10	110	100	no
Scan 11	110	100	0.25 mm Cu
Scan 12	110	100	0.25 mm Sn

The lamb bone was segmented from each µCT scan using watershed segmentation. The bone density was assigned based on the intensity values at each voxel, utilising the density calibration line calculated for each µCT scan, as described in Section 2.1.1. The mean and variance of the lamb femur's density distribution were computed from the density distribution histogram. A one‐way analysis of variance (ANOVA) was conducted using Minitab Statistical Software 21 (Minitab LLC. 2021, Minitab) to assess potential statistical significance among the mean densities of each CT scan, with a significance level set at 5%.

### 2.2 Cadaver Experiment Procedure: THA Surgical Process

Three healthy cadaveric femur samples, without any signs of arthritis or other pathological conditions, were obtained: a 70‐year‐old male (left), a 76‐year‐old female (right), and a 78‐year‐old male (right). The samples were thawed from −20°C to room temperature 24 h before the study. Approval was obtained from the Biomedical & Scientific Research Ethics Committee (BSREC) at the University of Warwick (Ref: BSREC 66/22‐23) and Research and Development, University Hospitals Coventry and Warwickshire (UHCW) NHS Trust (Ref: GF0503).

**Figure 1:**
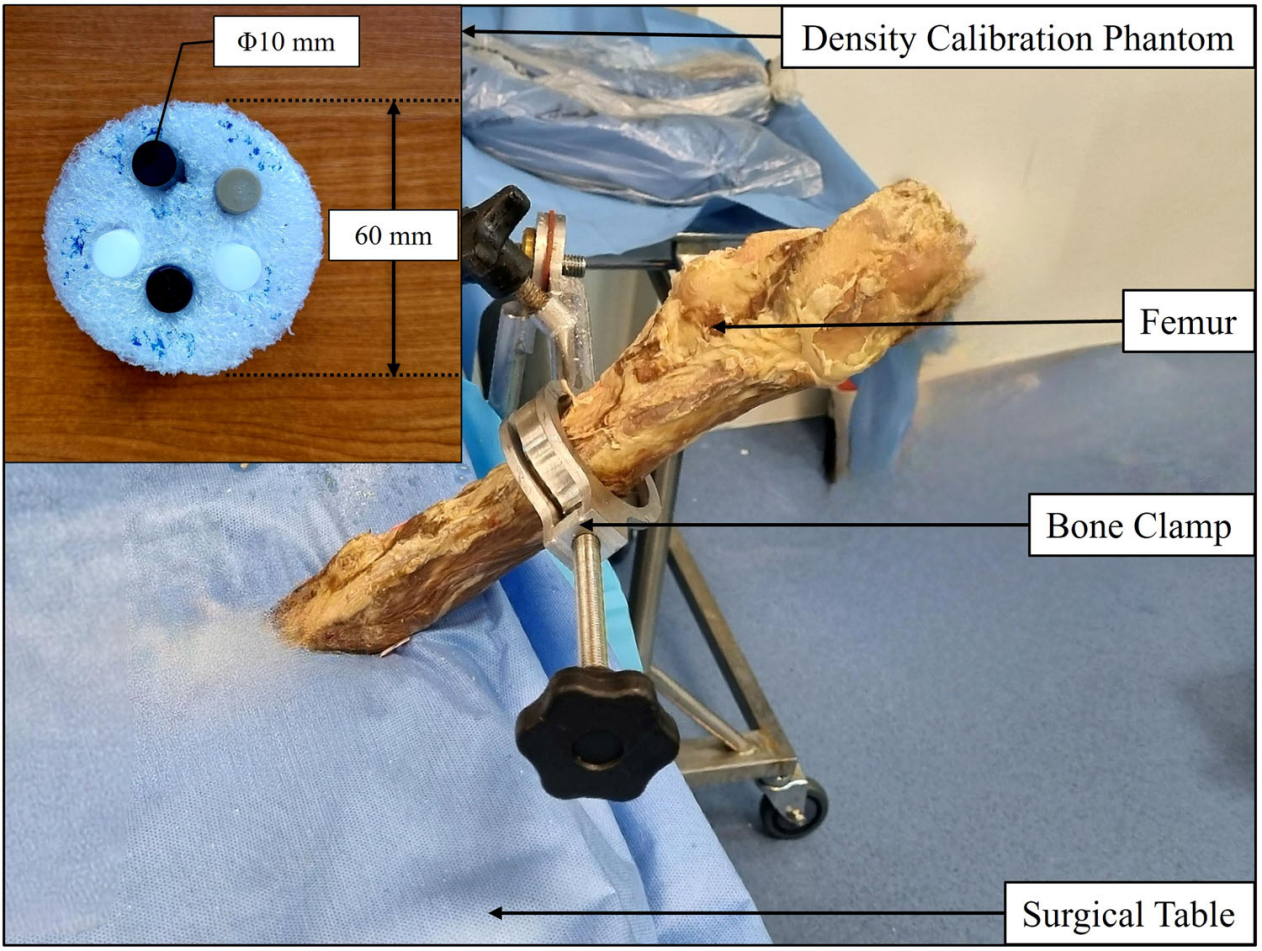
Hip arthroplasty experiment setup after broaching operation.

Initially, medical‐CT scans were conducted at University Hospitals Coventry and Warwickshire (UHCW) NHS Trust using a GE Medical Systems Revolution CT scanner (120 kV) with a voxel size of 0.5 × 0.5 × 0.625 mm. After bisecting each femur with the distal part removed, μCT scans were performed at the CiMAT μCT scanning centre on a Tescan Unitom XL scanner. Following the initial medical‐CT scans and μCT scans, THA was performed on the three femur samples by an experienced orthopaedic surgeon. The CT images were used to determine the appropriate size of the broach and implant needed for each femur sample. The femur samples were secured to a height‐adjustable surgical table using a bone clamp during the surgery. The orientation of the femurs was adjusted to replicate the actual surgical positioning. A neck osteotomy was performed, and the entry point into the femoral cavity was established at the piriformis muscle insertion, followed by insertion of a smooth intramedullary rod according to the surgical technique manual provided by the implant manufacturer (Corin, Cirencester, Gloucestershire, UK). The femoral cavity was prepared by compacting the trabecular bone with the Metafix compaction broach (Corin, Cirencester, Gloucestershire, UK). The size of the broach was incrementally increased until achieving the necessary longitudinal and rotational stability, as determined by the surgeon. Subsequently, the final broach was removed, and μCT scans were conducted using the Tescan Unitom XL to capture the bone geometry post‐compaction broaching. Figure 1 illustrates the experimental setup used for performing the THA of each femur sample after broaching operation and the in‐house DCP. After the second set of μCT scans, appropriately sized uncemented Metafix implants (Corin, Cirencester, Gloucestershire, UK) were implanted into each femur sample, matching the final broach size used. Finally, a set of μCT scans was performed on the Tescan Unitom XL to capture the bone geometry with the inserted uncemented implant. All the CT scans were performed in conjunction with the in‐house DCP, and a calibrated dimensional phantom was scanned after each μCT scan to calibrate the dimensional measurements [21]. The μCT scan parameters used for the three sets of μCT scans are listed in Table 2. Different scan parameters were utilised in the μCT scans of the bone samples to achieve optimal image quality by minimising noise and maximising contrast. After the implant was introduced post‐implantation, a higher voltage setting was necessary to ensure adequate X‐ray penetration through both the implant and the surrounding bone.

**Table 2:** μCT scan parameters.

Scan	Surgical step	Voltage (kV)	Power (W)	Exposure (ms)	Voxel size (μm)	Number of projections	Filter	Detector FOV (mm)
1	Pre‐surgery	100–130	70	156–232	70	3083	0.25 mm Cu	430 × 430
2	Post broaching	100	70	232–235	70	3683–4283	0.25 mm Cu	430 × 430
3	Post implantation	160	70	675–680	70	2933–3083	0.75 mm Sn	430 × 430

### 2.3 Determination of Change in Bone Density Due to THA

The cadaveric femurs were segmented from the CT scan data using watershed‐based segmentation in Avizo 3D 2021, and the bone density was assigned, as discussed in Section 2.1.1. After segmenting the femurs, the femur coordinate system (FCS) was defined according to the ISB recommendations [22, 23, 24]. The four CT scans of each femur (pre‐surgery medical‐CT, pre‐surgery μCT, post‐broaching μCT, and post‐implantation μCT) were aligned using best‐fit registration to evaluate the density change in a specific region and transfer the region of interest (ROI) between the scans.

For a quantitative comparison of bone density predicted from the different CT scans, the Gruen zones [25] were defined using the post‐implantation μCT scans and subsequently transferred to the other CT scans (pre‐surgery medical‐CT, pre‐surgery µCT, and post‐broaching µCT) after alignment. An additional ROI was defined for the μCT scans around the bone‐implant interface by setting a 1 mm thick ROI around it. This thickness was selected based on visual inspection to ensure it captured all broken trabecular bone debris. The 1 mm depth also aligns with literature, where densification around the interface has been observed up to this depth [13]. Subsequently, this ROI was transferred to the other two μCT scans (pre‐surgery µCT, and post‐broaching µCT). The intention of this step was to compare the alteration in bone volume fraction (BV/TV) resulting from the surgical intervention, specifically the ratio of bone volume (BV) to the total volume (TV) in the ROI around the bone‐implant interface.

## 3 Results

### 3.1 In‐House DCP Development and Validation

The densities of the DCP inserts measured are shown in Table 3, alongside the manufacturer‐specified density and densities obtained through mass and volume measurements using laser scan and µCT scan. The average density of the measured values was used as the density of the inserts to map the intensities. The measured density of the inserts was slightly different from the manufacturer‐specified values.

**Table 3:** DCP insert information.

		Measured					
	Manufacturer		Volume (mm^3^)		Density (g/cc)		
Material	Density (g/cc)	Mass (g)	Laser scan	CT scan	Laser scan	CT scan	Mean
Nylon	1.14	10.25 ± 0.0001	8925.62 ± 0.35	8863.84 ± 68.52	1.15 ± 0.009	1.16 ± 0.009	1.15
PEEK	1.32	10.87 ± 0.0001	8265.49 ± 0.74	8286.66 ± 22.71	1.32 ± 0.004	1.31 ± 0.004	1.31
Acetal	1.42	11.98 ± 0.0001	8444.13 ± 0.39	8391.10 ± 6.82	1.42 ± 0.001	1.43 ± 0.011	1.42
PPS	1.64	14.12 ± 0.0001	8593.82 ± 0.63	8808.03 ± 70.59	1.64 ± 0.012	1.6 ± 0.011	1.62
PTFE	2.18	18.21 ± 0.0001	8414.74 ± 0.28	8546.67 ± 40.47	2.16 ± 0.010	2.13 ± 0.012	2.15

**Figure 2:**
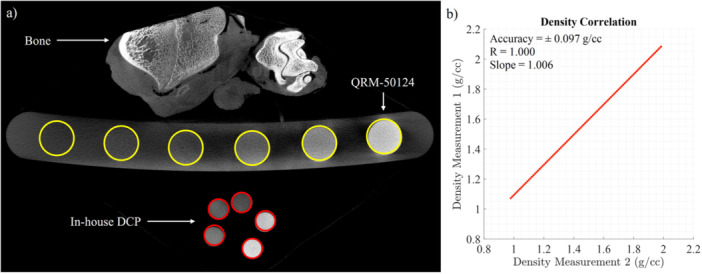
Density prediction comparison between using in‐house DCP and commercial DCP: (a) µCT image with the DCP inserts highlighted in red for in‐house DCP and yellow for QRM‐50124, (b) Correlation between the two density measurements of the bone.

Figure 2 shows the comparison between the density predictions using the in‐house DCP (measurement 1) and the commercial DCP QRM‐50124 (measurement 2). The results show a strong agreement between the density predictions obtained with the in‐house DCP (measurement 1) and the commercial DCP QRM‐50124 (measurement 2) as observed from Figure 2b. The accuracy of the density prediction using the in‐house DCP was determined to be ±0.097 g/cc (Figure 2b), assuming the commercial DCP QRM‐50124 as the reference. A strong linear correlation (*R* = 1) was observed between the two sets of density measurements, demonstrating that the in‐house DCP can reliably predict bone density (Figure 2b). Additionally, the slope of 1.006 indicates that the density values measured by the in‐house DCP were nearly identical to those measured by the commercial DCP (Figure 2b). The precision of the density prediction using the in‐house DCP was found to be ±0.052 g/cc, based on the analysis of 70 million data points from two consecutive µCT scans conducted under the same conditions, as shown in Figure 2a, without any external interference.

### 3.2 Density Prediction Sensitivity

**Figure 3:**
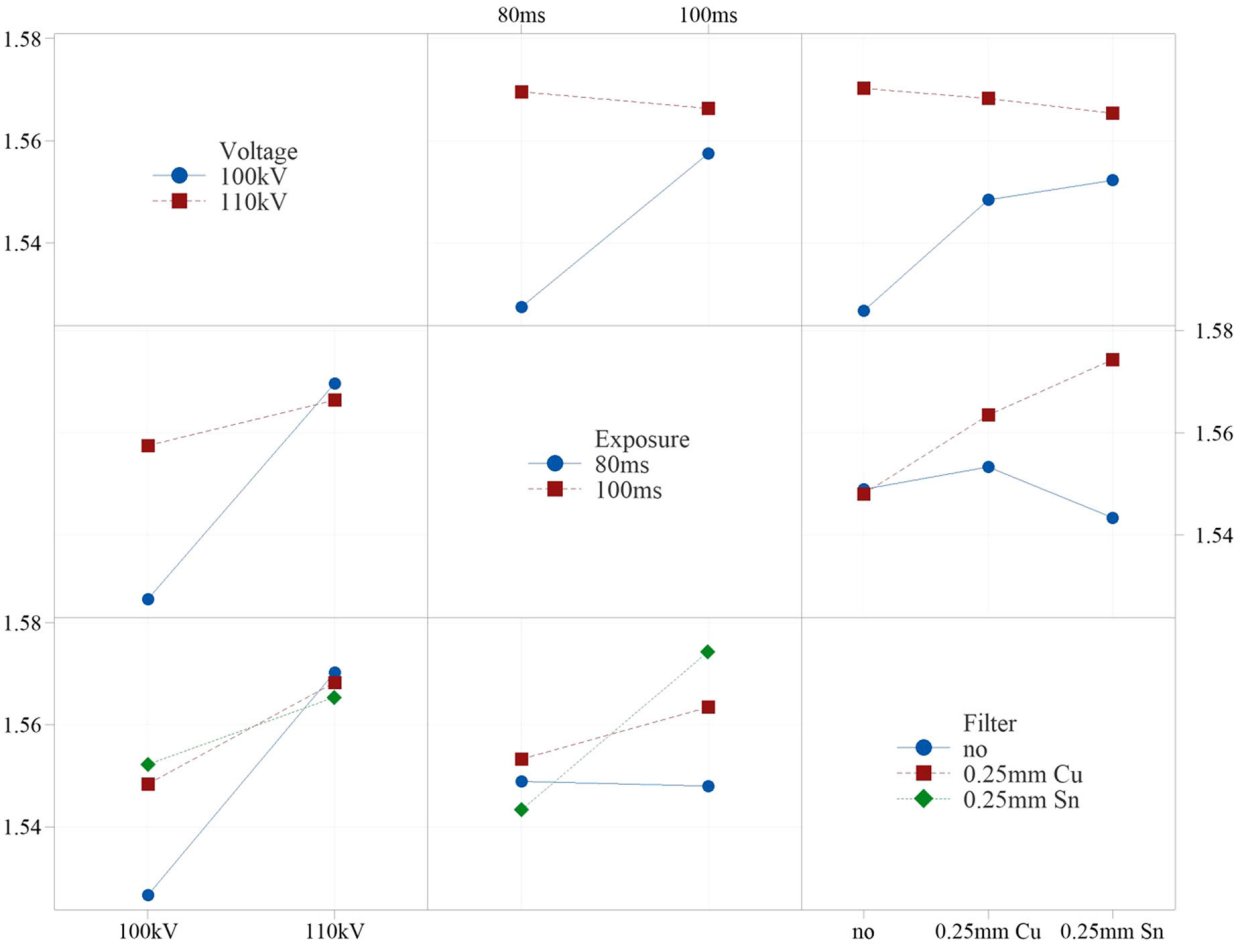
Sensitivity study factorial plots: Interaction plots due to change in scan parameters.

The sensitivity study on density prediction due to changes in µCT scan parameters revealed minimal variance in the mean density of the lamb femur, calculated at ±0.022 g/cc (σ), as observed in Table 4. It was found that 99% of the density variation could be attributed to these parameter alterations, with the variation falling within the ±0.129 g/cc (6σ) range (Table 4). The density mapping process appears to be largely unaffected by changes in the scan parameters, indicating that the method is robust with respect to variations in the µCT scan parameters. As shown in the factorial plot in Figure 3, the change in mean density remained within 6σ, further supporting this robustness.

**Table 4:** Density prediction sensitivity due change in CT scan parameters.

				Density (g/cc)
Scan	Voltage (kV)	Exposure (ms)	Filter	Mean
Scan 1	100	80	no	1.528
Scan 2	100	80	0.25 mm Cu	1.530
Scan 3	100	80	0.25 mm Sn	1.525
Scan 4	100	100	no	1.526
Scan 5	100	100	0.25 mm Cu	1.567
Scan 6	100	100	0.25 mm Sn	1.580
Scan 7	110	80	no	1.570
Scan 8	110	80	0.25 mm Cu	1.577
Scan 9	110	80	0.25 mm Sn	1.562
Scan 10	110	100	no	1.570
Scan 11	110	100	0.25 mm Cu	1.560
Scan 12	110	100	0.25 mm Sn	1.569
		Mean density	σ	0.022 g/cc
			6σ	0.129 g/cc

### 3.3 Medical‐CT and μCT Density Prediction Comparison

**Figure 4:**
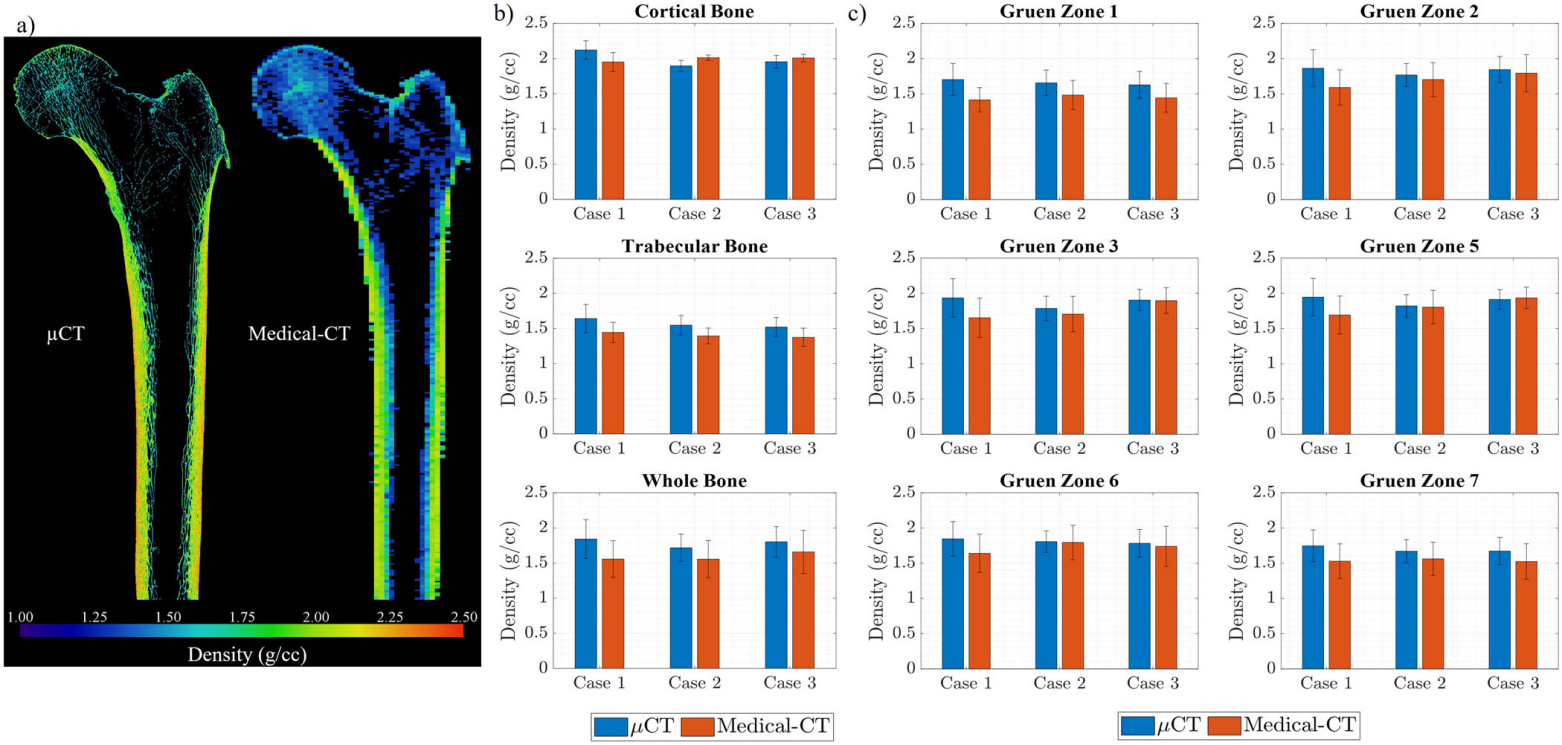
μCT and medical‐CT density comparison for the three femur samples: (a) Qualitative density comparison on a coronal plane for Case 1; (b) Comparison of different bone structures; (c) Comparison in the Gruen zones.

The comparison of density predictions between μCT and medical‐CT scans indicates that the bone density measured by the medical‐CT scan was consistently lower than that obtained from the μCT scans, as illustrated in Figure 4. This difference is particularly evident in Figure 4a, where the trabecular bone region in the femoral head and the femoral cavity show distinct colours. In the μCT scan, the bone appears shaded in greenish tones, while in the medical‐CT scan, it is shaded in bluish tones. This color difference reflects the lower density observed in the medical‐CT scan, as indicated by the colour map legend (Figure 4a). The difference in bone density between the two CT modalities was 0.196 ± 0.077 g/cc, as measured across the three femur samples (Figure 4b). This difference was particularly notable in the trabecular bone region, where the average density difference was nearly three times higher than in the cortical bone region (Figure 4b). However, in the cortical bone, the density values from both CT modalities were quite similar. Figure 4c presents a comparison of femur densities across the entire bone constituents, including both trabecular and cortical bone, in various Gruen zones. In areas with less trabecular bone, such as in Case 2 within Gruen zone 5, the density differences between the two scanning methods were minimal (Figure 4c). It should be noted that the ‘cases’ refer to different femur specimens.

### 3.4 Density Change Due to Surgical Intervention

**Figure 5:**
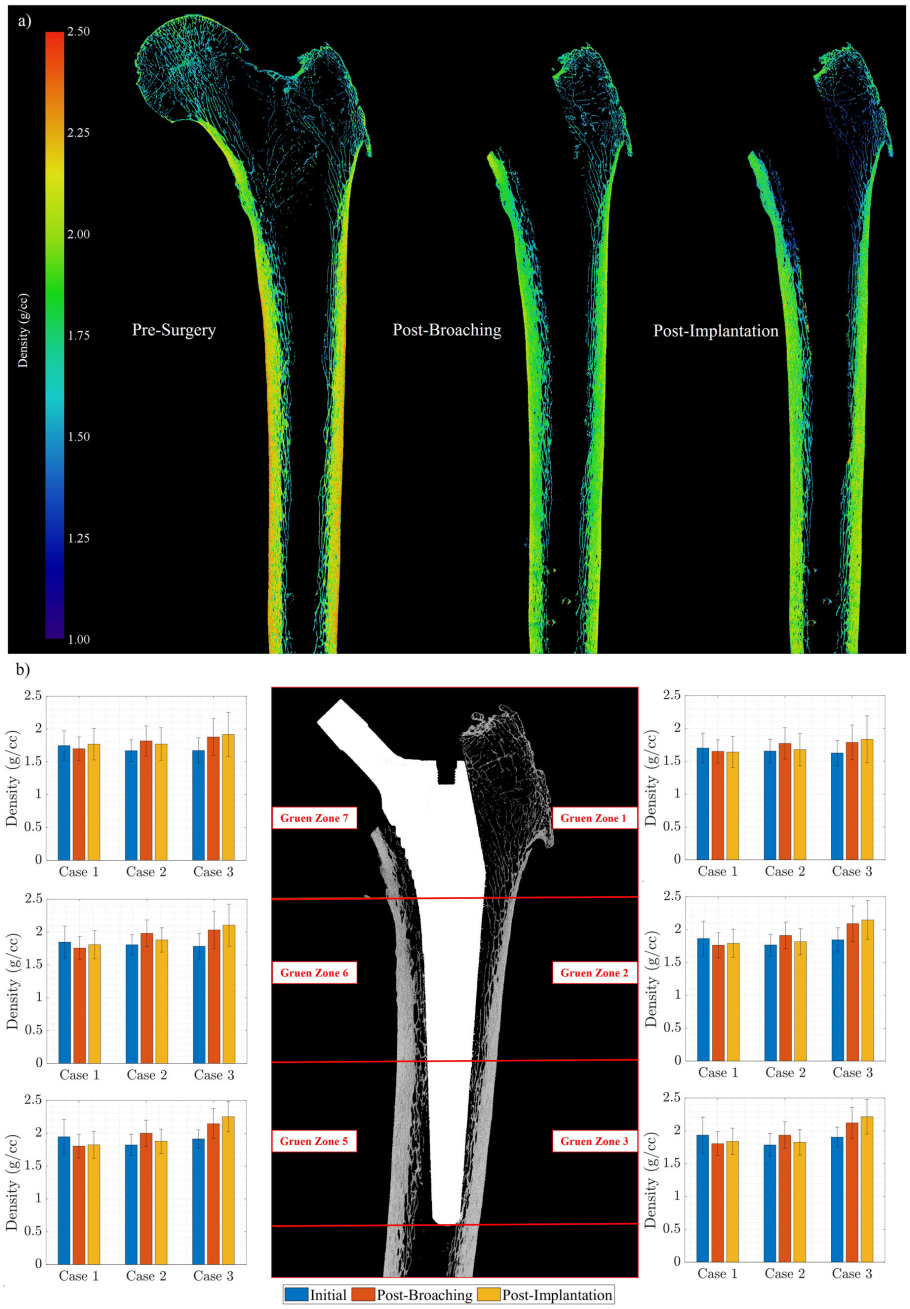
Bone density changes due to uncemented THA: (a) Qualitative comparison of bone density for Case 1, (b) Quantitative comparison across different Gruen zones for the three femur cases.

The change in bone density across the intermediate surgical stages—pre‐surgery, post‐broaching, and post‐implantation—is depicted in Figure 5. In Case 1, the outer surface of the cortical bone appeared denser before surgery compared to post‐broaching and post‐implantation, as observed from the colourmap in Figure 5a, where more regions are shaded in red. This observation is supported by a slight reduction in bone density, quantitatively shown across different Gruen zones in Figure 5b. However, this pattern was not consistent across the other two cases. In most Gruen zones, there was a slight increase in bone density after broaching and implantation compared to the pre‐surgery μCT scans (Figure 5b). Additionally, there was an increase in bone fraction (BV/TV) around the bone‐implant interface, ranging from 3.31% to 20.69%. This increase can be attributed to the accumulation of trabecular bone debris caused by the broaching process and uncemented implantation.

## 4 Discussion

In this study, the change in bone density due to the broaching operation and implantation during uncemented THA was investigated through a density mapping procedure using µCT. First, a robust method was established for the development of the in‐house DCP and the corresponding density mapping procedure through detailed validation and sensitivity studies. The validation study of the bone density predictions using the in‐house DCP, in comparison to a commercial DCP (QRM‐50124), showed a density prediction accuracy of ±0.097 g/cc and a precision of ±0.052 g/cc. Furthermore, the sensitivity of the density prediction to the µCT scan parameters was ±0.022 g/cc. Second, the density predictions using the density mapping procedure from the two CT scan modalities, namely μCT and medical‐CT, were investigated to assess the potential usefulness of the DCP in a clinical setting. Density comparisons between medical‐CT and μCT scans showed excellent agreement, especially in cortical bone. Finally, the change in bone density resulting from the broaching operation and uncemented implantation of the femur was assessed by μCT scanning of the femur at intermediate surgical stages on three cadaveric femur samples. An increase in bone density was observed compared to the density of the femurs following the broaching operation and implantation, with an average increase of 0.137 g/cc.

The commercially available DCPs are expensive and are often designed to be used with either medical‐CT scans or μCT scanners, mainly due to the dimensions and the base material of the DCP. Furthermore, the inserts of commercial DCPs often contain hydroxyapatite to mimic bone composition, which raises the cost of the DCP. Therefore, in this study, an in‐house DCP was developed specifically tailored for use in both medical‐CT and μCT scanners, with DCP insert densities ranging from 1.15 g/cc to 2.25 g/cc made from polymers in a very cost‐effective way. The validation study demonstrated the robustness of the density prediction using the in‐house DCP with an accuracy of ±0.097 g/cc and a precision of ±0.052 g/cc. Furthermore, the sensitivity of the density prediction to the µCT scan parameters was ±0.022 g/cc. Therefore, developing an in‐house DCP tailored for specific purposes, by validating insert densities and following the density mapping procedure described in this study, is feasible and cost‐effective compared to purchasing a commercial DCP, which tends to be orders of magnitude more expensive. Furthermore, the inclusion of hydroxyapatite in the DCP inserts might not be necessary, as observed from the results of the validation study. Using polymer inserts does not limit their maximum density, allowing for more accurate density predictions through interpolation. This improves upon the use of commercially available DCP inserts, which often require extrapolation when measuring cortical bone density (typically between 1.6 g/cc and 2 g/cc). Since the density of cortical bone usually exceeds that of DCP inserts (which have a maximum density of approximately 1.6 g/cc to 1.8 g/cc), extrapolation becomes necessary with DCP inserts. This will allow volumetric bone density to be used as an additional parameter for evaluating bone quality, alongside the DEXA scan, which provides areal bone density and is considered the gold standard for this measurement [26] and assessing fracture risk using FRAX [27]. Furthermore, incorporating patient‐specific volumetric bone density as an input parameter in FEA would enable personalised evaluations, helping predict potential surgical complications such as peri‐prosthetic fractures (PPF) and bone ingrowth.

A high degree of similarity in bone density between the medical‐CT and μCT scans was observed in both qualitative and quantitative comparisons across different bone structures and in various Gruen zones. The only noticeable difference was attributed to the resolution limitation of the medical‐CT scan, which prevented differentiation of trabecular bone microstructure. This discrepancy was particularly evident in quantitative comparisons within trabecular bone, where the average difference was ±0.147 g/cc, compared to ±0.054 g/cc in cortical bone. It can be concluded that for applications in which trabecular bone plays a crucial role, such as evaluating the primary stability of implants, the density predicted by medical‐CT scans may result in incorrect information. Consequently, in applications like finite element modeling of bone, where inhomogeneity and bone density are critical for mapping material constants, the trabecular bone density predicted by the medical‐CT scanner might lead to less accurate results [28]. The density predicted from the medical‐CT scan would provide a better estimate of the apparent density of the bone, which includes hydrated tissue mass by total specimen volume (bone + soft tissue + voids) [29], as the voxel volume from the medical‐CT scan would also encompass soft tissue and voids due to its lower resolution. On the other hand, the density predicted from the μCT scan would offer a more accurate estimate of the real density, which is hydrated tissue mass divided by bone tissue volume [29], since the voxel volume from the μCT scan primarily encompasses only bone and no soft tissue, benefiting from its higher resolution.

An increase in bone density was observed as a result of the broaching operation and implantation for the three femur cases in most of the ROIs, with the average increase in bone density being 0.137 g/cc across the three cases. The increase in bone density was within a similar range to that reported in the literature, which indicated an increase ranging from 0.16 g/cc to 0.30 g/cc [14, 16]. However, the densities reported in the literature consistently appeared to be slightly higher. This discrepancy in higher density reported at the bone‐implant interface in the literature could be attributed to the use of low‐resolution medical‐CT scans for quantifying the bone densification caused by the accumulation of trabecular bone debris during broaching. Medical‐CT scans have limitations in properly resolving trabecular bone, as the debris size falls below the minimum resolution achievable by the medical‐CT scan. Consequently, the bone appears denser in the medical‐CT scan, as each voxel near the bone‐implant interface is filled with more bone debris. This finding was corroborated by the current study, especially when comparing the change in bone fraction (BV/TV) due to the surgical intervention using μCT scan. An increase in bone fraction ranging from 3.31% to 20.69% in the ROI near the bone‐implant interface was observed, which is attributed to the accumulation of trabecular bone debris. This increase in bone volume fraction could potentially enhance the primary stability of the implant by acting as an autograft. Additionally, the accumulation of bone debris would increase bone‐implant contact, thereby promoting osseointegration through bone ingrowth after surgery [30]. As a result, this might discourage surgeons from flushing the bone debris after the broaching operation, potentially improving bone fixation. However, this claim has not been substantiated in this study, and further research is needed to account for other contributing factors. Furthermore, the density of the femur among the three cases predicted from either of the CT scan modalities using the in‐house DCP was 1.842 ± 0.276 g/cc. This finding aligns with previously reported femoral densities in the literature, which typically range between 1.1 g/cc and 2.0 g/cc [18, 19, 20, 31, 32, 33, 34].

The study presented has a few limitations. First, THA was performed on extracted femurs with a mechanical set‐up, which may not fully represent the actual surgical process. However, during broaching and uncemented implantation, the femurs were oriented to closely mimic actual surgery. Second, only one type of broach and implant was used, potentially making the results specific to this particular orthopaedic implant. Different sizes of broaches and implants were used in the three femur samples to minimise this limitation. Third, only three femur samples were used in this study. therefore, no statistical conclusions can be drawn on subject‐related variations. However, we do not expect significant changes in the results or conclusions with the inclusion of more samples, as the findings from the three femur samples were consistent. Future studies should consider these limitations to better understand the impact of surgical intervention on bone density.

## 5 Conclusion

The change in bone density due to the broaching operation and uncemented implantation, two major surgical steps of uncemented THA, was investigated for the first time using cadaver hip specimens and µCT scans. An in‐house DCP was developed cost‐effectively and validated against the predicted density of lamb bone using a commercial DCP. This led to a bone density prediction accuracy of ±0.097 g/cc, and a precision of ±0.052 g/cc using in‐house DCP in comparison with the results predicted using the commercial DCP. The sensitivity of the density measurement to the µCT scan parameters was ±0.022 g/cc. Density prediction of the cadaver femur using medical‐CT and µCT scans showed excellent agreement, particularly in cortical bone. However, the average difference in trabecular bone measurements was nearly three times higher, primarily due to the limitations of medical‐CT scans in resolving trabecular microstructure. The broaching and implantation processes resulted in an increase in bone density in the cadaveric femur, with an average increase of 0.137 g/cc. This increase was attributed to the accumulation of bone debris around the bone‐implant interface, leading to a rise in the bone volume fraction from 3.31% to 20.69%.

## Author Contributions

**Vineet Seemala:** conceptualisation, formal analysis, investigation, methodology, visualisation, original draft writing. **Mark A. Williams:** conceptualisation, methodology, supervision, review and editing. **Richard King:** conceptualisation, investigation, methodology, supervision, review and editing. **Sofia Goia:** investigation, review and editing. **Paul F. Wilson:** investigation, review and editing. **Arnab Palit:** conceptualisation, investigation, methodology, supervision, review and editing. All authors approved the final submitted manuscript.

## Conflicts of Interest

The authors declare that they have no known competing financial interests or personal relationships that could have appeared to influence the work reported in this paper.
